# Parliament and the Profession

**Published:** 1917-11-24

**Authors:** 


### Parliament and the Profession.

### Contagious Diseases Acts.

The Under-Secretary of State for War stated that he was not aware of any intention of the Army Council to make use of the Defence of the Realm Act for the purpose of reintroducing any provision which will have the same effect as the Contagious Diseases Acts.

### Detention of Venereal Diseases Patients.

The President of the Local Government Board having been asked if power could be given to the local assistance authority to detain cases of venereal disease when medically certified to be dangerous to others, replied that the question had been fully considered and it was not proposed to introduce legislation on the subject, it being felt that compulsory detention would deter many persons from seeking treatment and would do more harm than good.

### Medical Students.

The Minister of National Service (Sir Auckland Geddes) stated that the outlook with regard to the supply of medical men, and the question of military service as it affects medical students, has been under his careful review. The general policy adopted is to aim at maintaining a minimum output of 1,000 newly-qualified medical men per annum. This year no improvement can be made. In 1918 and 1919 the output will be below the desired minimum, though, he hoped, approaching it. Arrangements have been made that students serving in the Army who have completed two years' medical studies, and who had begun their studies prior to August 1, 1914, may claim to be returned to their studies.

### Child Mortality.

In response to an inquiry by Mr. Anderson as to the number of children dying every week whose death is due to preventable causes, said by Lord Rhondda to be at least 1,000 weekly, the President of the Local Government Board stated that in 1916 the deaths of children under five years of age registered in England and Wales was 109,966, although it is, of course, not possible to state exactly how many of these were due to preventable causes. Mr. Hayes Fisher expressed the opinion that if the local authorities in England and Wales were given the same powers as are now possessed by local authorities in Scotland and Ireland, and if they used them as he had reason to know they would do, infant welfare work could be developed so as to effect a great reduction in the number of deaths not only of infants but of mothers.

### Shell-Shock Patients and Air-Raids.

Colonel Clive asked the Under-Secretary of State for War whether it is possible to move the shell-shock patients at the 4th London General Hospital, Denmark Hill, and at Golders Green to a quieter area in view of the possible aggravation of their condition by air-raids. In respect of the patients at Denmark Hill Mr. Macpherson said that it may be possible to discontinue the neurological centre in London as a treatment centre in connection with changes which are now being made.

### The Ministry of Health and Infant Mortality.

Several questions having been addressed to the President of the Local Government Board with the apparent object of discovering the nature of the opposition to the proposed Ministry of Health, Mr. Fisher said that his Department were anxious to promote legislation with a view to secure for the local authorities in England and Wales the same powers in regard to maternity and infant welfare work as are already possessed by local authorities in Scotland and Ireland. Opposition had emanated from a body who described themselves as representing the various types of approved societies and the insurance committees of England and Wales, who desired to deal with the larger question of a Ministry of Public Health. The Local Government Board were of opinion that the gaining of these extra powers would enable local authorities to make rapid and vigorous use of them and would not in any way prejudice the ultimate consideration of the larger question of the Ministry of Health.

